# Conduit dynamics and post explosion degassing on Stromboli: A combined UV camera and numerical modeling treatment

**DOI:** 10.1002/2016GL069001

**Published:** 2016-05-28

**Authors:** T. D. Pering, A. J. S. McGonigle, M. R. James, G. Tamburello, A. Aiuppa, D. Delle Donne, M. Ripepe

**Affiliations:** ^1^ Department of GeographyUniversity of SheffieldSheffieldUK; ^2^ Sezione di PalermoIstituto Nazionale di Geofisica e VulcanologiaPalermoItaly; ^3^ Lancaster Environment CentreLancaster UniversityLancasterUK; ^4^ DiSTeMUniversità di PalermoPalermoItaly; ^5^ Dipartimento di Scienze della TerraUniversità di FirenzeFlorenceItaly

**Keywords:** Strombolian eruptions, gas slugs, uv cameras, gas flux, daughter bubbles, computational fluid dynamics

## Abstract

Recent gas flux measurements have shown that Strombolian explosions are often followed by periods of elevated flux, or “gas codas,” with durations of order a minute. Here we present UV camera data from 200 events recorded at Stromboli volcano to constrain the nature of these codas for the first time, providing estimates for combined explosion plus coda SO_2_ masses of ≈18–225 kg. Numerical simulations of gas slug ascent show that substantial proportions of the initial gas mass can be distributed into a train of “daughter bubbles” released from the base of the slug, which we suggest, generate the codas, on bursting at the surface. This process could also cause transitioning of slugs into cap bubbles, significantly reducing explosivity. This study is the first attempt to combine high temporal resolution gas flux data with numerical simulations of conduit gas flow to investigate volcanic degassing dynamics.

## Key Points

- Stromboli gas flux data suggest that trains of daughter bubbles may follow gas slugs
- CFD models suggest a complex relationship between daughter bubble production rate and Nf
- Bubble production can result in significant mass loss from ascending slugs

## 1 Introduction

Strombolian explosions are the archetypal events which occur every few minutes [*Ripepe et al*., 2002] at Stromboli volcano (Aeolian Islands, Italy) and are thought to result from the ascent and bursting of gas slugs [e.g., *Chouet et al*., 1974; *Blackburn et al*., 1976; *Seyfried and Freundt*, 2000, and references therein]. Thus, the study of slug flow dynamics is pivotal to understanding the processes driving Strombolian eruptions, and analytical and computational fluid dynamic (CFD) models have been developed to help interpret geophysical signals [e.g., *James et al*., 2004, 2008; *O*'*Brien and Bean*, 2008; *Del Bello et al*., 2012].

With the advent of ultraviolet (UV) cameras [e.g., *Mori and Burton*, 2006; *Bluth et al*., 2007; *Tamburello et al*., 2011], gas fluxes can now be measured at sufficiently high temporal resolutions to also provide insight into slug fluid dynamics. Such data have recently enabled the identification of gas flux coda for the first time, which are periods of elevated emissions following individual Strombolian events [*Tamburello et al*., 2012]. Coda are potentially produced from trains of “daughter bubbles” that have been released from, and subsequently follow, the base of ascending slugs. Here we use CFD models of slug ascent and daughter bubble production as a first step to quantify this process and assess its wider implications for conduit fluid dynamics.

The morphology and behavior of ascending gas slugs depend on parameters which include conduit radius, magma density, and magma viscosity. Notably, magma viscosity and conduit radius also play key roles in determining the form and the degree of turbulence in the wakes that follow slugs [e.g., *Campos and Guedes de Carvalho*, 1988]. For long slugs, wake form has been shown to be a function of the dimensionless inverse viscosity, *N*_*f*_, which represents a ratio of external forces to internal viscous forces:

(1) $$N_{f}=\;\frac{\rho_{m}}{\mu }\sqrt{g{\left(2r_{c}\right)}^{3}}\text{,}$$

where *ρ*_*m*_ is the liquid density, *μ* is liquid viscosity, *g* is acceleration due to gravity, and *r*_*c*_ is tube radius [*Campos and Guedes de Carvalho*, 1988; *Nogueira et al*., 2006]. For *N*_*f*_ values < 500 slug wakes are closed (recirculatory) and axisymmetric, while for values > 500, symmetry is lost and wakes become increasingly open and turbulent *Nogueira et al*., 2006]. With sufficient wake turbulence, gas can be removed from the main slug by bubbles being sheared from its base [*Wallis*, 1969; *Campos and Guedes de Carvalho*, 1988]. The resulting daughter bubbles can then either become entrained within the slug wake and subsequently reincorporated back into the slug [*Campos and Guedes de Carvalho*, 1988] or can be ejected from the wake, to rise independently from, and behind, the main slug [e.g., *Bouche et al*., 2010]. To the authors' knowledge, *Fernandes et al*. [1983] detail the only previous attempt to quantify gas mass loss from the base of slugs but did not consider the subsequent escape of the gas from the wake.

In the volcanic literature, daughter bubble production has been discussed briefly [e.g., *James et al*., 2006; *Suckale et al*., 2010; *Llewellin et al*., 2014], but the implications for Strombolian eruptions have yet to be quantified. Using values of 2700 kg m^3^ for magma density [e.g., *Vergniolle and Brandeis*, 1996; *Métrich et al*., 2001], 200–500 Pa s^−1^ for magma viscosity [*Vergniolle et al*., 1996], and 1–3 m for conduit radius [e.g., *Harris and Stevenson*, 1997; *Delle Donne and Ripepe*, 2012] to represent the system at Stromboli, equation (1) gives *N*_*f*_ values of 47–621, suggesting that daughter bubble production could be possible. Therefore, motivated by the observed gas coda signals in UV camera measurements of eruptions at Stromboli, we report here on the use of CFD models to explore the potential effects of daughter bubble generation on Strombolian eruptions. This is the first reported attempt to combine high temporal resolution volcanic gas flux data with numerical simulations of gas flow in conduits to investigate the dynamics of volcanic degassing.

## 2 UV Camera Measurements of SO_2_ Flux

**Figure 1:**
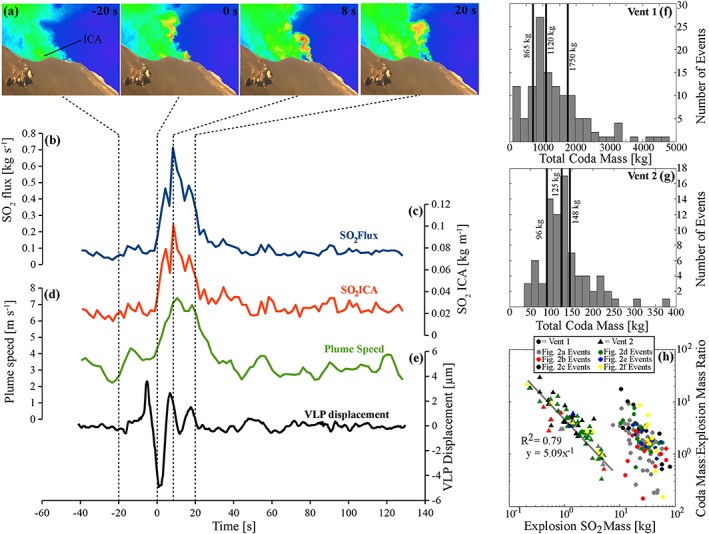
(a) A sequence of UV camera images showing relative concentration of SO_2_, with line (at −20 s) showing where integrated column amount (ICA) is calculated; (b) example SO_2_ flux, (c) ICA of SO_2_, (d) plume speed, and (e) VLP displacement for a single Vent 2 event. (f and g) The distribution of coda masses for Vent 1 and Vent 2 events, respectively. (h) The relationship between SO_2_ mass and coda:explosion mass ratio.

We first characterized and quantified the gas release from a total of 200 events, which were categorized into two classes: Vent 1 events, numbering 120, which arose from the craters (mostly the south east crater; SEC), and Vent 2 events, numbering 80 and emanating from a hornito on the edge of the SEC. Vent 1 events were relatively ash free, included the ejection of bombs, and were associated with very long period (VLP) seismic signals, while Vent 2 events released gas only and did not always produce a VLP signal (see Figure 1e for VLP during a Vent 2 event). Events of both types occurred every ≈2–10 min, with impulsive gas release lasting ≈10–30 s in each case. The gas flux measurements were performed on 11, 13, 21, 22, 23, and 25 June, and 3 July 2014, using the permanent UV camera network installed by the Università di Palermo and Università di Firenze, within the framework of the FP7‐ERC project “BRIDGE.” The cameras imaged the entire crater terrace, and gas fluxes were determined ≈50 m above the vents.

Figure 1 shows example integrated column amounts of SO_2_ and gas velocities (calculated using optical flow algorithms [e.g., *Horn and Schunck*, 1981; *Peters et al*., 2015]), which were used to calculate SO_2_ fluxes. The data used to characterize each event were taken from the base of initial flux peak until measured flux values returned to background levels. SO_2_ masses for the explosions were calculated by integrating underneath the peaks of each flux trace associated with the initial impulsive gas release (see Movie S1 and Text S2 for further details) and, for coda masses, until flux levels returned to background levels; see Text S2 for examples (also see Figure 1, *Kantzas et al*. [2010], and *Tamburello et al*. [2011, 2012], and references therein, for full details on UV camera calibration and image processing).

**Figure 2:**
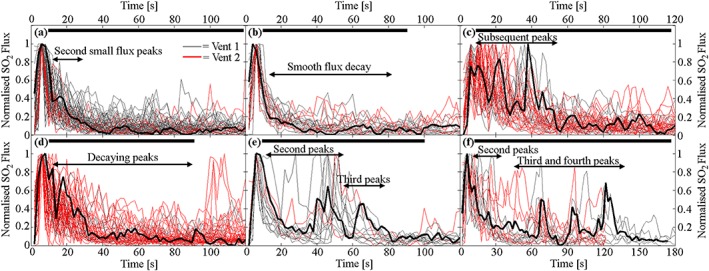
Graphs (a–f) summarizing the characteristic degassing signatures observed following all Vent 1 and Vent 2 events. All events have been normalized by subtracting the minimum value and dividing by the maximum. The black horizontal bar at the top of the plots represents maximum event duration, while the thick black line traced on all subfigures represents a characteristic event. See supporting information Table S6 for details on how events were categorized.

To facilitate comparison of all measured Vent 1 and 2 events, the explosive traces were normalized by subtracting each event's minimum value and then dividing by the maximum. The normalized data show a range of characteristics, and events can be visually separated into a number of distinct families (Figure 2). Each event shows an initial peak in flux, followed by a flux coda [e.g., *Tamburello et al*., 2012]. Additional observed features include a variable number of subsequent flux peaks within the coda, of differing magnitudes and coda durations.

In general, SO_2_ masses were an order of magnitude larger for Vent 1 events than for the Vent 2 events, in both the main explosion (Figure 1h) and coda (Figures 1f and 1g) components (see Table S1 in the supporting information for further detail and for total gas masses calculated using the gas chemical compositions from *Burton et al*. [2007]). The latter compositional data pertain to Vent 1 type activity alone; therefore, the Vent 2 total masses must be considered best estimates on the basis of available data. For the Vent 2 events, with increasing explosion mass, a higher proportion of the overall released mass is contained within the explosion (Figure 1h). For Vent 1 events, a similar relationship exists, but this appears somewhat skewed by events with rather low coda masses (Figure 1h).

## 3 CFD Modeling of Slug Flow and Gas Flux

To explore the potential for daughter bubble production as a source for gas coda, slug ascent was simulated using the computational fluid dynamics package Ansys Fluent®. A series of 3‐D models were constructed based on a quarter pipe section (e.g., as in *James et al*. [2008]) uniformly meshed with 0.05 m^3^ to 0.1 m^3^ cells (adjusted to keep computation times reasonable and small enough to resolve the falling film thickness). In each case, the volume‐of‐fluid method, with implicit body force enabled, was employed in a pressure‐based solver, and thermal effects were also simulated. An explicit time‐stepping method was used with the time‐step fixed between 10^−2^ and 10^−3^ s, and a maximum Courant number of 0.25. A constant pressure upper boundary condition was used to represent the conduit at a depth of ≈100 m within the magma (2,750,025 Pa), and a zero‐flow bottom boundary condition was imposed. Two fluids were simulated: magma (with temperature of 1000°C [e.g., *Harris and Stevenson*, 1997], density of 2700 kg m^3^ [e.g., *Métrich et al*., 2001], and surface tension of 0.4 N m^2^) and water vapor (for the slug, with thermal conductivity of 0.0261 W m^−1^K^−1^ and a ratio of specific heats of 1.4). Water vapor is the only compressible phase within the model and was chosen for the slug composition, as it is the dominant species in the volcanic gas chemistry (with 83% of the molar weight [*Burton et al*., 2007].

Ansys Fluent® has previously been shown capable of modeling full 3‐D slug flow and daughter bubble production [e.g., *Taha and Cui*, 2006; *Araújo et al*., 2012] at engineering scales. To provide confidence in our quarter pipe model, a number of validation runs were performed (TC1–TC5) to ensure that it replicated the five regimes of behavior reported in *Taha and Cui* [2006] over *N*_*f*_ values of 84–1528. To reproduce the conditions of *Taha and Cui* [2006], these simulations used a pipe of radius 9.5 mm and length 190 mm, with liquids of surface tension 0.064 N m^2^, densities ranging 1129 to 1223 kg m^−3^, and kinematic viscosities 9.7 × 10^−5^ to 5.47 × 10^−6^ m^2^ s^−1^. Dry air was used for the slug. Supporting information Movie S2 demonstrates our model validation by showing matching wake features (i.e., an increasingly closed wake on decreasing *N*_*f*_ value and a commensurate decrease in turbulence in the wake area) and onset of daughter bubble production for each of the *N*_*f*_ values used by *Taha and Cui* [2006].

Following validation, 3‐D Stromboli‐specific model runs (S1–S12) were implemented for a range of magma viscosities (200, 300, 400, and 500 Pa s^−1^) and conduit radii (1, 2, and 3 m), i.e., *N_f_* = 48–621 [e.g., *Vergniolle et al*., 1996; *Harris and Stevenson*, 1997; *Delle Donne and Ripepe*, 2012]. The initial slug and conduit lengths were set to ≈2.5 and 10 times the conduit radius, respectively (e.g., for *r_c_* = 2 m, the conduit length was 20 m). These parameters were chosen to allow the flow regime to develop while minimizing compute time, and by simulating slugs at depths ≈100 m, the effects of gas expansion did not substantially contribute to liquid motions. The magma density and viscosity values chosen here represent bulk column values; deviations from these values could occur through the presence of bubbles and crystals, with an increase in either of these acting to reduce *N_f_*. The models were run in parallel on the University of Sheffield Linux high‐performance computing cluster, with a total simulation time of <2 weeks.

**Figure 3:**
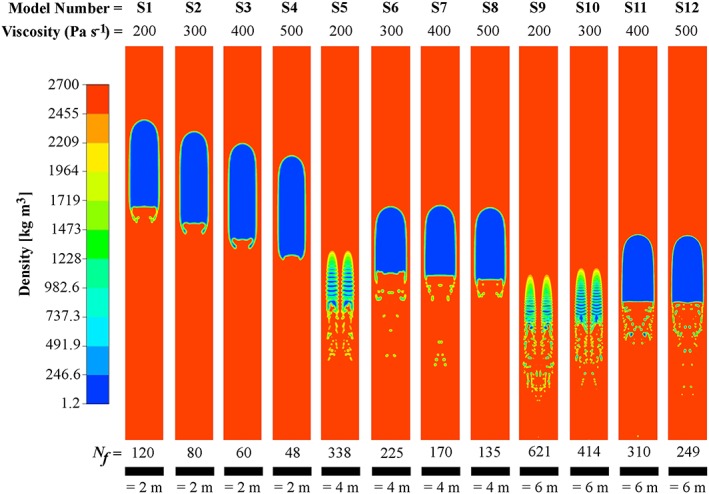
(a) Example slug morphologies from the series of 3‐D simulations (S1–S12). All models were captured at 10 s from initiation. Daughter bubble production is observed in several of the model runs.

**Figure 4:**
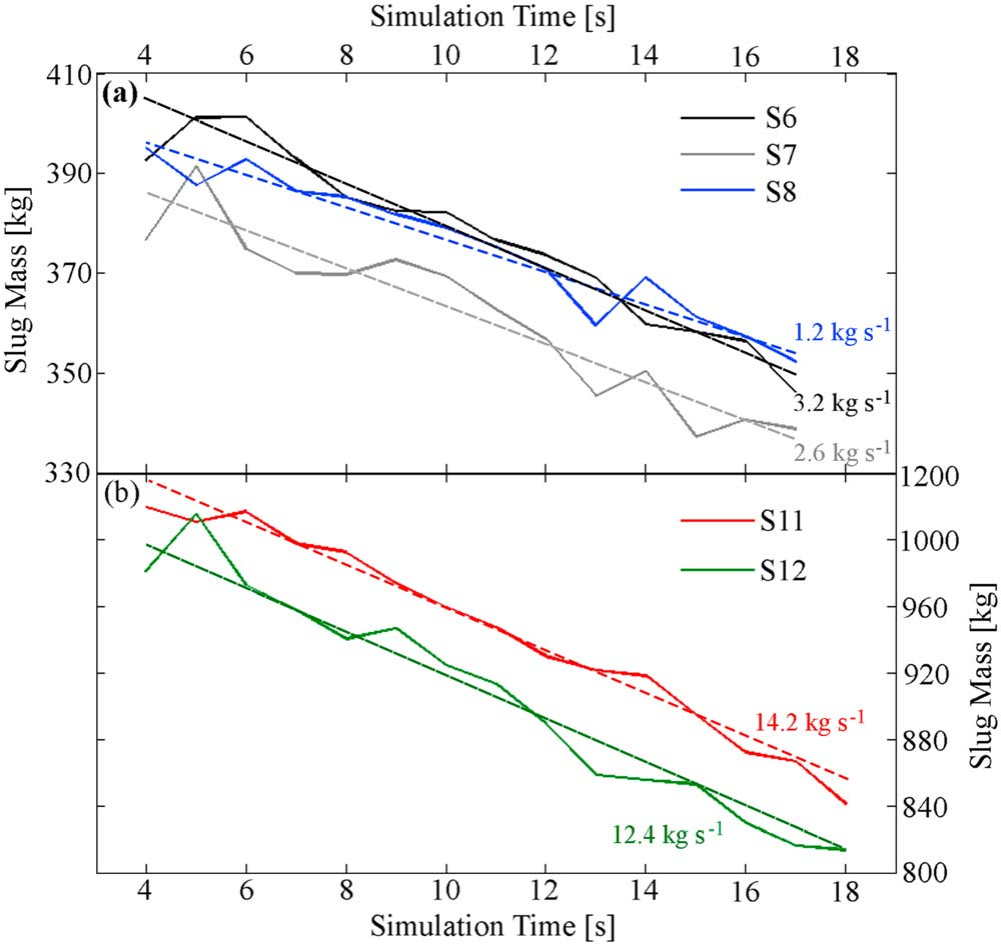
Slug gas mass during daughter bubble producing model runs. (a) Model runs S6, S7, and S8 with *r*_*c*_ = 2 m. (b) S11 and S12 with *r*_*c*_ = 3 m. Dashed lines indicate linear best fit representing average mass loss rates (plotted against *N_f_* in Figure 5a).

**Figure 5:**
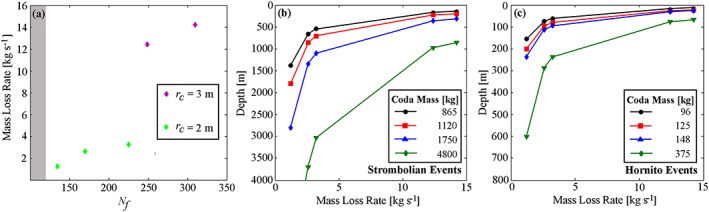
(a) The relationship between gas mass lost from slugs per second and the dimensionless inverse viscosity, *N*_*f*_, for daughter bubble producing model runs shaded region represents *N*_*f*_ values for simulations which did not produce daughter bubbles. (b and c) The depths from which a slug must rise to generate coda masses representing the 1st quartile, median, 3rd quartile, and maximum values of the measured distributions at Stromboli (Figures 1f and 1g), for Vent 1 and Vent 2 events, respectively.

In five of these models, the production of daughter bubbles (see Table S2 for summary) was observed from the base of the rising slugs, to varying degrees (Figure 3 and supporting information Movie S3). These mass loss rates are stable for the duration of simulations, after a short initial start‐up transient (Figure 4), and ranged between ≈1.2 and 14.2 kg s^−1^ between the different models (Figure 5a). For *N*_*f*_ values >330 (models S5, S9, and S10), slugs were observed to separate into four individual bubbles on initiation (see Figure 3); this occurred for slugs that were wider than the maximum bubble diameter determined by theoretical considerations of bubble nose instability [*Suckale et al*., 2010] and is likely to be an artifact of the transient and unphysical conditions at model initiation (i.e., static fluid surrounding a stationary bubble). The possibility that daughter bubble production was triggered by similar transients in other models was tested by simulating a stable, non‐daughter bubble producing slug (at *N*_*f*_ ≈ 100, with *μ* = 1200 Pa s and *r_c_* = 3 m) for ≈ 10 s (i.e., so that a flow regime had fully developed), then decreasing the magma viscosity to 400 Pa s, to give *N*_*f*_ = 310. This resulted in a transition to daughter bubble production with rate similar to that generated if the model had originally been started with *N*_*f*_ = 310 (model S11).

Onset of daughter bubble production in our Stromboli models occurred at lower *N*_*f*_ values than in our validation models and in previous work [e.g., *Nogueira et al*., 2006; *Taha and Cui*, 2006]. The differences between the simulations are dominated by scale (from mm to m), surface tension (0.064 N m^2^ and 0.4 N m^2^), and dynamic viscosity (2 to 3 orders of magnitude difference). It is possible therefore that *N_f_* does not fully capture all the physics required to parameterize daughter bubble production, and caution is required if extrapolating laboratory‐scale results to volcanic scenarios.

## 4 Discussion

All the events measured at Stromboli had gas coda of ≈20–160 s in duration, which in some cases contained post‐explosive peaks in emission (e.g., Figures 2c–2f). As Stromboli persistently degasses, it is conceivable that the coda could result from interactions between the rising slug and gas already contained within the conduit [*Gaudin et al*., 2014]; for example, a pressure drop in the magma below a gas slug could allow additional gas exsolution in the wake region. If slugs are produced from regions of foam collapse or ascending pockets of gas saturated magma, bubble trains could also potentially be produced from “leftover” bubbles following slug formation. Variability and flux peaks within coda could potentially be caused by the close rise and burst of independent bubbles or slugs, by gas dispersal processes in the upper conduit and atmosphere, or by complexities in slug burst due to heterogeneities in magma rheology [*Del Bello et al*., 2015; *Capponi et al*., 2016]. This said, given the efficiency of gas transfer from slugs into daughter bubbles demonstrated within our simulations, we do suggest that daughter bubble production could play a key role in driving coda generation in Strombolian volcanism.

Our field measurements show that coda gas masses are ≈53–75% of total event gas masses (i.e., explosion plus coda) for Vent 1 events, and ≈70–84% for Vent 2 events (Figure 1, using data between the first and third quartiles of the distribution). Given the range of mass loss rates calculated from the simulations, ≈ 1.2–14.2 kg s^−1^, it is entirely plausible that coda on the order of those observed in our field measurements could be generated. We note that the gas mass loss rates for the Stromboli simulations do not show a straightforward relationship with *N_f_* (Figure 5a), further suggesting that work is warranted to characterize the processes behind slug mass loss rates. Future 3‐D models will also enable inclined conduit scenarios to be investigated, which would be of particular relevance to Stromboli, where the conduit is at 40° from the vertical [*Chouet et al*., 2003].

Daughter bubble production has interesting volcanological implications beyond the production of gas coda. Under conditions of sufficiently high *N_f_* for daughter bubble production and shedding from the wake region, rising slugs will effectively dissipate during ascent. In this case, slugs may only substantially occur in the shallow portions of a conduit, where decompressive gas expansion can outweigh volumetric loss from daughter bubble production. At greater depths, ascending slug‐sized gas pockets will dissipate until they transition to a stable morphology, such as cap bubbles, at which point the effective increase in falling film thickness and decrease in ascent velocity will reduce wake turbulence and hence mass loss [*Wallis*, 1969]. Furthermore, the release of daughter bubbles would add significant numbers of smaller bubbles to the conduit, some of which would coalesce (e.g., see models S7 and S12 in the supporting information Movie S2) and, over greater flow distances than modeled here, could generate larger bubbles, providing an explanation for the post‐explosive peaks in the flux data (Figure 2). If slugs are sufficiently large, and a constant slug mass loss rate during ascent is assumed, then coda gas masses could be used to infer initial slug depths (Figures 5b and 5c). If measured coda masses were generated from the smaller gas mass loss rates calculated in the simulations, then (using slug rise speeds of ≈1.97 – 2.54 m s^−1^, see supporting information S1 for details) slugs could ascend from previously suggested formation depths of ~3 km [*Burton et al*., 2007; *Chouet et al*., 2008] while larger mass loss rates would limit slug flow to depths < 1000 m (Figure 5b). The Vent 2 coda masses are suggestive of much shallower source depths, as indicated in Figure 5c, with the higher mass loss rates corresponding to depths < 100 m. This analysis, along with other characteristic gas mass differences between Vent 1 and Vent 2 events (Figure 1h), suggests that the gas slugs driving each process may arise from different source regions or processes. However, the strong event variability, particularly for the Vent 1 explosions (Figures 1g, and h), precludes more detailed interpretation from the current data set, and we encourage coda measurement to be integrated into longer‐term monitoring campaigns, with a view to substantially increasing the number of observations.

## 5 Conclusions

Using UV camera measurements of SO_2_ gas fluxes from Strombolian explosions, we demonstrate that a significant proportion of gas from each event can be contained within a flux coda that follows the initial explosively erupted gas mass. We suggest, supported by evidence from CFD simulations, that the observed flux coda could be generated by the production of daughter bubbles and the formation of a daughter bubble train that follows a rising slug. The production of daughter bubbles is demonstrated to vary with the inverse viscosity of the system and potentially with conduit radius. Slugs of insufficient initial mass can dissipate into cap bubbles and a trailing bubble train prior to reaching the surface. This allows for the intriguing possibility that, in some systems, slug morphology can only be sustained in near‐surface regions where depressurisation‐driven gas expansion [*James et al*., 2008] exceeds gas volume loss to daughter bubbles.

## Supporting information



Supporting Information S1

Table S4

Table S5

Movie S1

Movie S2

Movie S3

## References

[grl54463-bib-0001] Araújo, J. D. P. , J. M. Miranda , A. M. F. R. Pinto , and J. B. L. M. Campos (2012), Wide‐ranging survey on the laminar flow of individual Taylor bubbles rising through stagnant Newtonian liquids, Int. J. Multiphase Flow, 43, 131–148.

[grl54463-bib-0002] Blackburn, E. A. , L. Wilson , and R. S. J. Sparks (1976), Mechanisms and dynamics of Strombolian activity, J. Geol. Soc., 132, 429–440, doi:10.1144/gsjgs.132.4.0429.

[grl54463-bib-0003] Bluth, G. J. S. , J. M. Shannon , I. M. Watson , A. J. Prata , and V. J. Realmuto (2007), Development of an ultra‐violet digital camera for volcanic SO_2_ imaging, J. Volcanol. Geotherm. Res., 161, 47–56.

[grl54463-bib-0004] Bouche, E. , S. Vergniolle , T. Staudacher , A. Nercessian , J.‐C. Delmont , and M. Frogneux (2010), The role of large bubbles detected from acoustic measurements on the dynamics of Erta 'Ale lava lake (Ethiopia), Earth Planet. Sci. Lett., 295, 37–48, doi:10.1016/j.epsl.2010.03.020.

[grl54463-bib-0005] Burton, M. , P. Allard , F. Muré , and A. La Spina (2007), Magmatic gas composition reveals the source depth of slug‐driven Strombolian explosive activity, Science, 317, 227–230. 17626881 10.1126/science.1141900

[grl54463-bib-0006] Campos, J. B. L. M. , and J. R. F. Guedes de Carvalho (1988), An experimental study of the wake of gas slugs rising in liquids, J. Fluid Mech., 196, 27–37.

[grl54463-bib-0007] Capponi, A. , M. R. James , and S. J. Lane (2016), Gas slug ascent in a stratified magma: Implications of flow organisation and instability for Strombolian eruption dynamics, Earth Planet. Sci. Lett., 435, 159–170, doi:10.1016/j.epsl.2015.12.028.

[grl54463-bib-0008] Chouet, B. , N. Hamisevicz , and T. R. McGetchin (1974), Photoballistics of volcanic jet activity at Stromboli, Italy, J. Geophys. Res., 279(32), 4961–4976, doi:10.1029/JB079i032p04961.

[grl54463-bib-0010] Chouet, B. , P. Dawson , T. Ohminato , M. Martini , G. Saccorotti , F. Giudicepietro , G. De Luca , G. Milana , and R. Scarpa (2003), Source mechanisms of explosions at Stromboli Volcano, Italy, determined from moment‐tensor inversions of very‐long‐period data, J. Geophys. Res., 108(B1), 2019, doi:10.1029/2002JB001919

[grl54463-bib-0011] Chouet, B. , P. Dawson , and M. Martini (2008), Shallow‐conduit dynamics at Stromboli Volcano, Italy, imaged from waveform inversions, Geol. Soc. Spec. Publ., 307, 57–84.

[grl54463-bib-0012] Del Bello, E. , E. W. Llewellin , J. Taddeuicci , P. Scarlato , and S. J. Lane (2012), An analytical model for gas overpressure in slug‐drive explosions: Insights into Strombolian volcanic eruptions, J. Geophys. Res., 117, B02206, doi:10.1029/2011JB008747.

[grl54463-bib-0013] Del Bello, E. , S. J. Lane , M. R. James , E. W. Llewellin , J. Taddeucci , P. Scarlato , and A. Capponi (2015), Viscous plugging can enhance and modulate explosivity of Strombolian eruptions, Earth Planet. Sci. Lett., 423, 210–218, doi:10.1016/j.epsl.2015.04.034.

[grl54463-bib-0014] Delle Donne, D. , and M. Ripepe (2012), High‐frame rate thermal imagery of Strombolian explosions: Implications for explosive and infrasonic source dynamics, J. Geophys. Res., 117, B09206, doi:10.1029/2011JB008987.

[grl54463-bib-0015] Fernandes, R. C. , R. Semiat , and A. E. Dukler (1983), Hydrodynamic model for gas‐liquid slug flow in vertical tubes, AIChE J., 29(6), 981–989, doi:10.1002/aic.690290617.

[grl54463-bib-0016] Gaudin, D. , J. Taddeucci , A. Harris , T. Orr , M. Bombrun , and P. Scarlato (2014), When puffing meets Strombolian explosions: A tale of precursors and coda, EGU, Vienna General Assembly.

[grl54463-bib-0017] Harris, A. J. L. , and D. S. Stevenson (1997), Thermal observations of degassing open conduits and fumaroles at Stromboli and Vulcano using remotely sensed data, J. Volcanol. Geotherm. Res., 76, 175–198.

[grl54463-bib-0018] Horn, B. K. P. , and B. G. Schunck (1981), Determining optical flow, Artif. Intell., 17, 185–203.

[grl54463-bib-0019] James, M. R. , S. J. Lane , B. Chouet , and J. S. Gilbert (2004), Pressure changes associated with the ascent and bursting of gas slugs in liquid‐filled vertical and inclined conduits, J. Volcanol. Geotherm. Res., 129(1–3), 61–82.

[grl54463-bib-0020] James, M. R. , S. J. Lane , and B. A. Chouet (2006), Gas slug ascent through changes in conduit diameter: Laboratory insights into a volcano‐seismic source process in low‐viscosity magmas, J. Geophys. Res., 111, B05201, doi:10.1029/2005JB003718.

[grl54463-bib-0021] James, M. R. , S. J. Lane , and S. B. Corder (2008), Modelling the rapid near‐surface expansion of gas slugs in low‐viscosity magmas, Geol. Soc. Lond. Spec. Publ., 307, 147–167.

[grl54463-bib-0022] Kantzas, E. P. , A. J. S. McGonigle , G. Tamburello , A. Aiuppa , and R. G. Bryant (2010), Protocols for UV camera volcanic SO_2_ measurements, J. Volcanol. Geotherm. Res., 194, 55–60.

[grl54463-bib-0024] Llewellin, E. W. , M. Burton , H. Mader , and M. Polacci (2014), Conduit speed limit promotes formation of explosive ‘super slugs’, San Francisco, AGU General Assembly.

[grl54463-bib-0025] Métrich, N. , A. Bertagnini , P. Landi , and M. Rosi (2001), Crystallization driven by decompression and water loss at Stromboli volcano (Aeolian Islands, Italy), J. Petrol., 42, 1471–1490.

[grl54463-bib-0026] Mori, T. , and M. Burton (2006), The SO_2_ camera: A simple, fast and cheap method for ground based imaging of SO_2_ in volcanic plumes, Geophys. Res. Lett., 33, L24804, doi:10.1209/2006GL027916.

[grl54463-bib-0027] Nogueira, S. , M. L. Riethmuller , J. B. L. M. Campos , and A. M. F. R. Pinto (2006), Flow patterns in the wake of a Taylor bubble rising through vertical columns of stagnant and flowing Newtonian liquids: An experimental study, Chem. Eng. Sci., 61, 7199–7212.

[grl54463-bib-0028] O'Brien, G. S. , and C. J. Bean (2008), Seismicity on volcanoes generated by gas slug ascent, Geophys. Res. Lett., 35, L16308, doi:10.1029/2008GL035001.

[grl54463-bib-0029] Peters, N. , A. Hoffmann , T. Barnie , M. Herzog , and C. Oppenheimer (2015), Use of motion estimation algorithms for improved flux measurements using SO_2_ cameras, J. Volcanol. Geotherm. Res., 300, 58–69, doi:10.1016/j.volgeores.2014.08.031.

[grl54463-bib-0030] Ripepe, M. , A. J. L. Harris , and R. Carniel (2002), Thermal, seismic and infrasonic evidences of variable degassing rates at Stromboli volcano, J. Volcanol. Geotherm. Res., 118(3–4), 285–297.

[grl54463-bib-0031] Seyfried, R. , and A. Freundt (2000), Experiments on conduit flow and eruption behaviour of basaltic volcanic eruptions, J. Geophys. Res., 105(B10), 23,727–23,740, doi:10.1029/2000JB900096.

[grl54463-bib-0032] Suckale, J. , B. H. Hager , L. T. Elkins‐Tanton , and J.‐C. Nave (2010), It takes three to tango: 2. Bubble dynamics in basaltic volcanoes and ramifications for modelling normal Strombolian activity, J. Geophys. Res., 115, B07410, doi:10.1029/2009JB006917.

[grl54463-bib-0033] Taha, T. , and Z. F. Cui (2006), CFD modelling of slug flow in vertical tubes, Chem. Eng. Sci., 61(2), 676–687, doi:10.1016/j.ces.2005.07.022.

[grl54463-bib-0034] Tamburello, G. , E. P. Kantzas , A. J. S. McGonigle , and A. Aiuppa (2011), Recent advances in ground‐based ultraviolet remote sensing of volcanic SO_2_ fluxes, Ann. Geophys., 54(2), 199–208.

[grl54463-bib-0035] Tamburello, G. , A. Aiuppa , E. P. Kantzas , A. J. S. McGonigle , and M. Ripepe (2012), Passive vs. active degassing modes at an open‐vent volcano (Stromboli, Italy), Earth Planet. Sci. Lett., 359–360, 106–116.

[grl54463-bib-0036] Vergniolle, S. , and G. Brandeis (1996), Strombolian explosions: 1. A large bubble breaking at the surface of a lava column as a source of sound, J. Geophys. Res., 101(B9), 20,433–20,447, doi:10.1029/96JB01178.

[grl54463-bib-0037] Vergniolle, S. , G. Brandeis , and J. C. Mareschal (1996), Strombolian explosions 2. Eruption dynamics determined from acoustic measurements, J. Geophys. Res., 101(B9), 20,449–20,466, doi:10.1029/96JB01925.

[grl54463-bib-0039] Wallis, G. B. (1969), One‐Dimensional Two‐Phase Flow, McGraw‐Hill, New York.

