# Supplementary material for: Conduit dynamics and post explosion degassing on Stromboli: A combined UV camera and numerical modeling treatment
Source: Geophys Res Lett. 2016 May 28;43(10):5009–16. doi: 10.1002/2016GL069001 (PMC4950127; doi:10.1002/2016GL069001)
Supplement: Supplementary file 1 — Supporting Information S1 [file GRL-43-5009-s006.docx]

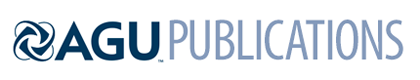


*Geophysical Research Letters*

Supporting Information for

**Conduit dynamics and post-explosion degassing on Stromboli: a combined UV camera and numerical modeling treatment**

Pering, T. D*^a^., McGonigle, A. J. S^a,d^., James, M. R^b^., Tamburello, G^c^., Aiuppa, A^c,d^. Delle Donne, D.^c^, Ripepe, M^e^.

^a^University of Sheffield, Dept. of Geography, Winter Street, S10 2TN, United Kingdom

^b^Lancaster Environment Centre, Lancaster University, Lancaster, LA1 4YQ, UK

^c^DiSTeM, Università di Palermo, via Archirafi, 22, 90123 Palermo, Italy

^d^Istituto Nazionale di Geofisica e Vulcanologia, Sezione di Palermo, Via Ugo La Malfa, 153, 90146, Palermo, Italy

^e^Dipartimento di Scienze della Terra, Università di Firenze, Via La Pira, 4, 50121, Firenze, Italy

**Contents of this file**

Text S1

Text S2

Tables S1 to S3

**Additional Supporting Information (Files uploaded separately)**

Captions for Tables S4 and S5

Captions for Movies S1 to S2

**Introduction**

In the supplementary information we provide additional data on the validation of our computational model developed in Ansys Fluent, and on the calculation of slug base rise speed used in the text (S1). In addition we provide movies of all of our simulations (Movies S2 and S3) to demonstrate the production of daughter bubbles and validation. All data referring to explosion and coda masses of both Vent 1 (Tables S3 and S4) and Vent 2 events (Tables S3 and S5) are also provided along with a Movie in S1. In S2 we describe and provide examples of explosion and coda SO_2_ mass calculation, we also describe how the events in Fig. 2 were categorized (Table S6).

S1.

The value for $N_{f}$ can be calculated using Eq. 1 defined in the text. Slug base rise speed ($u_{sl}$) is then calculated using the following (Viana et al. 2003):

$u_{sl}=Fr\sqrt{2gr_{c}}$,

$Fr=0.34\left[ 1+\left( \frac{31.08}{N_{f}} \right)^{1.45} \right]^{-0.71}$.

The parameters *g* and $r_{c}$, are gravitational acceleration (9.81 m s^-1^) and conduit radius.

Volume of the incompressible magma phase was conserved to <1% giving further confidence in our measurements. All compressible slug masses (kg H_2_O) were calculated using the ideal gas law, taking into account model outputs of pressure in the magma and magma temperature. At the initiation of each model there is a short period of stabilization (the model still converges) where the slug reaches a constant base velocity and achieves a constant film thickness (where indeed the slug behaves as a slug).

**S2.**

In Figure S1 we demonstrate the approach taken in the calculation of explosion and coda masses for three separate events. The impulsive initial release of gas from the slug is taken to be the explosive mass for each event, corresponding to the initial dominant flux peak(s); this typically lasted ≈ 10 – 20 s for each event, although ranged up to 30 s in some cases. Following this, the gas is assumed to be part of the coda until returning to background flux levels (i.e. that seen prior to each event and indicated by the black lines in Figure S1). Figure S2 shows a longer time period with multiple Vent 1 events, illustrating the relative stability of background flux levels. Table S6 describes the different categories, which were used in the production of Figure 2 within the text.

Here, out of necessity, we require definition of a clear demarcation between explosive and coda degassing for our analysis. This said, the burst process and emission of gas is demonstrably complex at Stromboli (e.g. Del Bello et al. 2015; Capponi et al. 2016), particularly given the possibility of alterations to the slug involved with the bursting process. There is therefore the possibility that the boundary between these two degassing regimes could be more complicated that supposed here. In this case, the treatment here must be taken to result in best estimates of gas masses associated with these two degassing regimes in the context of explosions on Stromboli, on the basis of our current empirical capability.

Figure S1. Three examples (a, b, and c) of SO_2_ mass calculation for explosion (red) and coda (blue) mass. The black line shows the background flux level used for integration purposes.


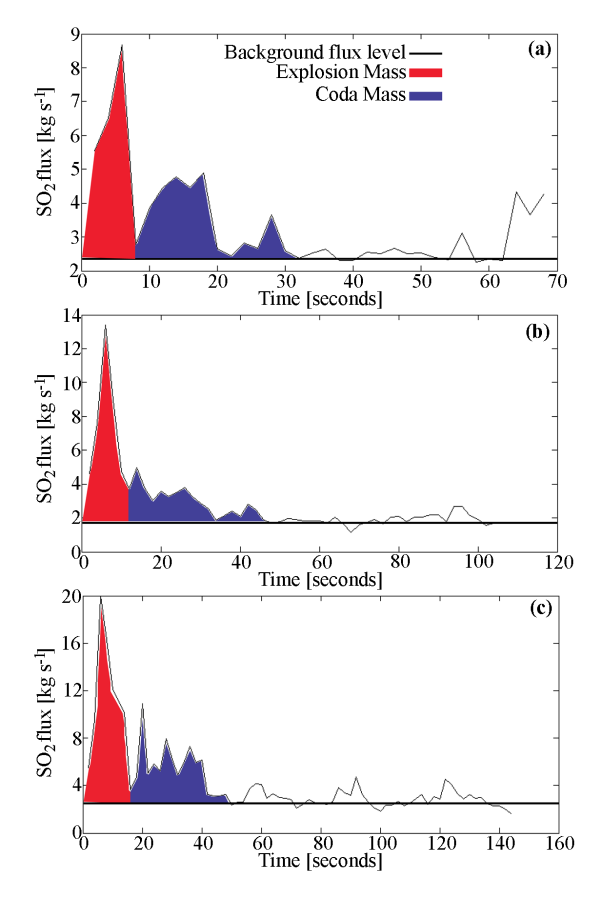


Figure S2. Example of a longer period of SO_2_ flux data used within this study.


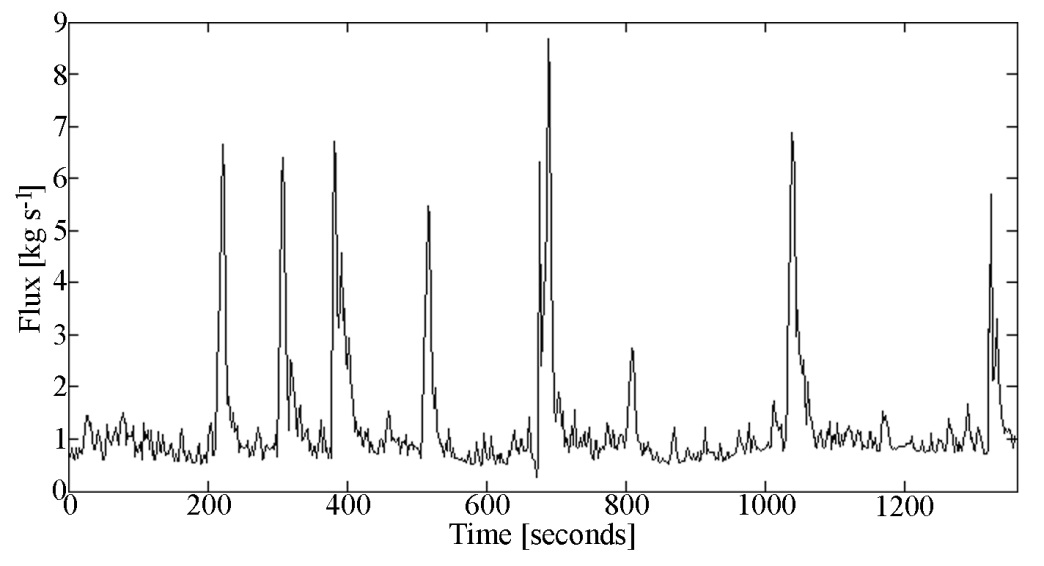


Table S1. Summary of parameters used in producing the Taha and Cui (2006) validation runs.

| Name | Radius (m) | Density (kg m^3^) | Kinematic Viscosity (m^2^ s) | *N_f_* |
| --- | --- | --- | --- | --- |
| TC1 | 0.0095 | 1223 | 9.7 x 10^-5^ | 84 |
| TC2 | 0.0095 | 1206 | 4.67 x 10^-5^ | 176 |
| TC3 | 0.0095 | 1202 | 4.0 x 10^-5^ | 205 |
| TC4 | 0.0095 | 1190 | 2.5 x 10^-5^ | 325 |
| TC5 | 0.0095 | 1129 | 5.47 x 10^-6^ | 1528 |

Table S2: Summary of Stromboli specific model runs, including initial parameters and final values for slug length, mass and overall mass lost per second.

| Name | Radius (m) | Viscosity (Pa s^-1^) | *N_f_* | 2D or 3D | Notes |
| --- | --- | --- | --- | --- | --- |
| S1 | 1 | 200 | 120 | 3D |  |
| S2 | 1 | 300 | 80 | 3D |  |
| S3 | 1 | 400 | 60 | 3D |  |
| S4 | 1 | 500 | 48 | 3D |  |
| S5 | 2 | 200 | 338 | 3D | Slug separates |
| S6 | 2 | 300 | 225 | 3D | Daughter bubbles |
| S7 | 2 | 400 | 170 | 3D | Daughter bubbles |
| S8 | 2 | 500 | 135 | 3D |  |
| S9 | 3 | 200 | 621 | 3D | Slug separates |
| S10 | 3 | 300 | 414 | 3D | Slug separates |
| S11 | 3 | 400 | 310 | 3D | Daughter bubbles |
| S12 | 3 | 500 | 249 | 3D | Daughter bubbles |

Table S3. Table showing statistics of SO_2_ masses of Vent 1 (strombolian) and Vent 2 (puffing) explosions and events. Also shown are the total gas masses for events determined using Burton et al. [2007] gas ratios.

|  | Minimum | Mean | Maximum |
| --- | --- | --- | --- |
| Vent 1 Explosion [kg SO_2_] | 8 | 30 | 82 |
| Vent 1 Event [kg SO_2_] | 18 | 87 | 225 |
| Vent 1 Explosion Total Gas Mass [kg] | 181 | 708 | 1,949 |
| Vent 1 Event Total Gas Mass [kg] | 428 | 2,072 | 5,360 |
| Vent 2 Explosion [kg SO_2_] | 0.2 | 1.9 | 5.3 |
| Vent 2 Event [kg SO_2_] | 2 | 7.5 | 19.6 |
| Vent 2 Explosion Total Gas Mass [kg] | 5 | 46 | 125 |
| Vent 2 Event Total Gas Mass [kg] | 47 | 179 | 467 |

Table S4. Table showing all of the Vent 1 explosion and coda masses.

Table S5. Table showing all of the Vent 2 explosion and coda masses.

Table S6. Table showing how each of the events were categorized and the number of events whether Vent 1 or Vent 2 in each section The event types refer to the Figure 2 subsections.

| **Event Type** | **Vent 1 Events** | **Vent 2 Events** | **Description** | **Event Time (s)** |
| --- | --- | --- | --- | --- |
| Type a | 53 | 7 | Flux peak with a smaller peak after ≈ 20 – 30 s. Coda demonstrates small variability following this. | 30 – 120 s |
| Type b | 16 | 6 | An initial flux peak, which decreases rapidly. Smooth flux decay over 30 – 90 s. | 30 – 90 s |
| Type c | 15 | 23 | Three initial peaks of varying magnitude ≈ 60 s following burst. Coda includes smaller variations. | 50 – 120 s |
| Type d | 13 | 35 | Peaks decaying in magnitude after the initial burst, shortest event duration of all types. | 30 – 60 s |
| Type e | 11 | 2 | One or two additional flux peaks following the initial burst occur, with these peaks occurring when flux levels almost return to background. | 60 – 100 s |
| Type f | 9 | 7 | Similar to Type a event, however, multiple flux peaks are observed between ≈ 60 – 140 s. | 120 – 180 s |

Movie S1. Movie showing UV camera and visible images of one Vent 1 event and one Vent 2 event.

Movie S2. Movie showing the results of all Taha and Cui (2006) validation model runs.

Movie S3. Movie showing the results of all 3D Stromboli specific model runs.

Supplementary References

Llewellin, E. W., E. Del Bello, J. Taddeucci, P. Scarlato, and S. J. Lane (2012), The thickness of the falling film of liquid around a Taylor bubble, *Proc. R. Soc. B, 468,* doi:10.1098/rspa.2011.0476.

Viana, F., R. Pardo, R. Yánez, J. L. Trallero, and D. D. Joseph (2003), Universal correlation for the rise velocity of long gas bubbles in round pipes, *J. Fluid Mech., 494,* 379–398.
